# Natural Compounds for Preventing Ear, Nose, and Throat-Related Oral Infections

**DOI:** 10.3390/plants10091847

**Published:** 2021-09-06

**Authors:** Adelina-Gabriela Niculescu, Alexandru Mihai Grumezescu

**Affiliations:** 1 Faculty of Engineering in Foreign Languages, University Politehnica of Bucharest, 060042 Bucharest, Romania; niculescu.adelina19@gmail.com; 2 Faculty of Applied Chemistry and Materials Science, University Politehnica of Bucharest, 060042 Bucharest, Romania; 3 Research Institute of the University of Bucharest—ICUB, University of Bucharest, 050657 Bucharest, Romania; 4 Academy of Romanian Scientists, 3 Ilfov Street, 50044 Bucharest, Romania

**Keywords:** oral health, oral infections, ENT infections, alternative treatments, natural compounds, natural antimicrobials

## Abstract

Oral health is an essential element in maintaining general well-being. By preserving the complex equilibrium within the oral microbial community, commensal microorganisms can protect against extrinsic pathogenic threats. However, when an imbalance occurs, the organism is susceptible to a broad range of infections. Synthetic drugs can be administered to help the body fight against the fungal, bacterial, or viral burden. Nonetheless, they may produce undesirable consequences such as toxicity, adverse effects, and drug resistance. In this respect, research has focused on finding safer and more efficient alternatives. Particularly, increasing attention has been drawn towards developing novel formulations based on natural compounds. This paper reviews the plant-based, algae-based, and beehive products investigated for their antimicrobial properties, aiming to thoroughly present the state of the art on oral infection prevention in the ear, nose, and throat (ENT) field.

## 1. Introduction

The oral microbiome is an essential component of the human microbial community, playing a vital role in protecting against the colonization of extrinsic microbes, which can affect overall health. In addition, the oral microbiome is associated with systemic diseases, as the mouth represents an entry point to both the respiratory and digestive systems, which are also highly vascularized [1,2,3].

An imbalance in the complex equilibrium between the various microorganisms from the healthy oral cavity is intimately connected to the pathogenesis and development of numerous oral and systemic diseases [2,4,5]. Furthermore, factors such as poor oral hygiene, trauma, malnutrition, use of antibiotics, wear of dentures, underlying medical conditions, and radio- or chemotherapy also contribute to the occurrence of oral infection [4,6,7].

Specific pathogens can overgrow and spread in the oral mucosae, extend to surrounding tissues, and, if left untreated, produce systemic infections [6,8]. To avoid such a cascade of events, preventing oral infections has become an intense research topic. Preventive medicine is mostly focused on reducing oral infections and their associated complications via good oral hygiene [6,9]. In this respect, mouth rinses and toothpaste usually contain active ingredients like chlorhexidine, hyaluronic acid, and fluorides, which, although effective, may present some clinical disadvantages (e.g., taste alterations, mouth dryness, tooth discoloration, calculus accumulation, mucosal lesions) [9]. What is more, the use of synthetic chemical antimicrobials has been shown to produce drug-resistant microorganism strains which are no longer affected by conventional treatments. Hence, there emerged the need to develop novel treatment options [10,11].

To overcome the issues associated with chemical products, natural compounds have attracted interest in preventing and treating oral infections [5,10,11,12]. In this respect, a broad range of natural sources has been investigated for their pharmacological properties to find better solutions for bacterial, fungal, and viral oral infections [13,14,15].

This review comprehensively presents the oral microbiome composition, its potential imbalances, and conventional treatments for the most frequent oral infections. A special focus is further given to natural antimicrobial compounds that are systematically described from the points of view of their sources and potential applications.

## 2. Oral Microbiome Composition

**Figure 1:**
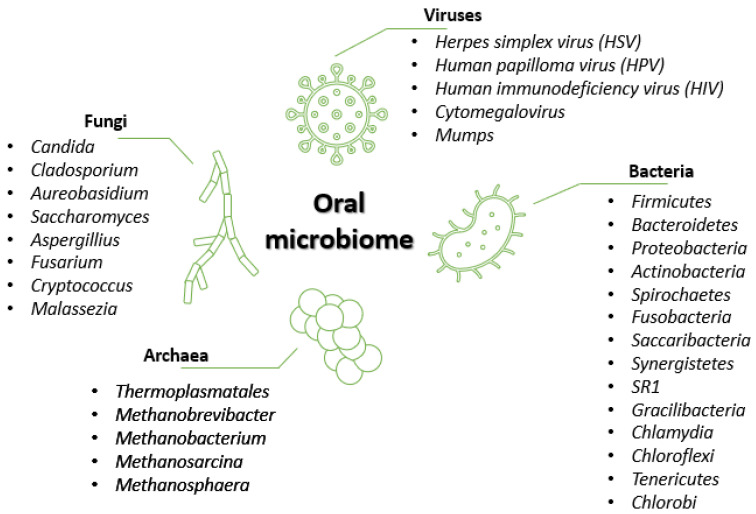
Oral microbiome composition. Created based on information from references in the literature [2,4,5,16,17].

The human oral cavity is colonized by diverse microbial flora, mainly comprised of bacteria, fungi, and viruses [16] (Figure 1).

Bacteria represent the predominant microorganism type, with the human oral cavity containing more than 500 different species [2,18]. According to the literature [2,4,18], 95% of the oral bacterial community belongs to 6 major phyla, namely, *Firmicutes*, *Proteobacteria*, *Actinobacteria*, *Bacteroidetes*, *Fusobacteria,* and *Spirochaetes*. The remaining 5% of the taxa comprise microorganisms from phyla like *Saccharibacteria*, *Synergistetes*, *SR1*, *Gracilibacteria*, *Chlamydia*, *Chloroflexi*, *Tenericutes*, and *Chlorobi* [2]. Some genera, such as *Streptococcus*, *Gemella*, *Granulicatella*, *Veillonella*, and *Fusobacterium*, inhabit almost all oral sub-niches, whereas other genera, such as *Prevotella*, *Bacteroides*, *Corynebacterium*, *Pasteurella*, and *Neisseria*, have been found in selected sites [4].

Despite being studied to a lesser degree as compared to bacteria, fungi are widely present in the oral cavity. They are usually reported as opportunistic pathogens in immunocompromised individuals, but fungal organisms also belong to the healthy oral microbiota, which includes up to 101 fungal species [2]. The most common genus is *Candida* (with *C. albicans* as the predominant species), followed by *Cladosporium*, *Aureobasidium*, *Saccharomyces*, *Aspergillus*, *Fusarium*, *Cryptococcus*, and *Malassezia* [2,4].

Archaea represents a minor part of the oral microflora, restricted to limited species such as *Thermoplasmatales*, *Methanobrevibacter*, *Methanobacterium*, *Methanosarcina*, and *Methanosphaera*. The prevalence and numbers of these methanogens are increased in periodontitis patients, but archaea can be found in healthy individuals as well [2].

Several viruses can also be present in the oral cavity, and can be involved in oral ulcers, oral tumors, oral infections, and periodontitis [16]. Except for herpes simplex virus (HSV) and cytomegalovirus, viruses appear to be oral transients, primarily affecting other body structures [19]. Unlike the other components of the oral microbiota, most viruses in the mouth are associated with diseases [2]. Viruses like HSV, human papillomavirus (HPV), human immunodeficiency virus (HIV), cytomegalovirus, and mumps are responsible for lesions inside and around the mouth, salivary gland infection, gingivostomatitis, papilloma, condylomas, focal epithelial hyperplasia, and more [2,16].

## 3. Oral Infections: Causative Pathogens and Aggravating Potential

As oral health implies the maintenance of a complex microbiotic equilibrium, fluctuations in the availability of oxygen, nutrients, and the pH-mediating effect of saliva can result in the growth of some microorganisms and further cause opportunistic infections [3,4,7,20]. Such changes may occur in immunosuppressed individuals, patients undergoing radiotherapy, chemotherapy, prolonged antimicrobial therapy, and steroid administration, and people suffering from xerostomia, diabetes, or cancer [3,7,20,21,22,23,24,25,26,27,28]. Nonetheless, otherwise healthy individuals may develop oral infections due to a series of risk factors such as smoking, alcohol consumption, poor nutrition, ill-fitting prosthesis, infancy, old age, or pregnancy [12,20,24,26,27,29].

The persistence of pathogens inside the mouth leads to focal infection points, which may further cause secondary health problems such as biofilm formation, difficulty in chewing and swallowing, altered taste sensation, halitosis, systemic malnutrition, and weight loss [28,30].

To particularize the discussion and better understand how such infections may aggravate, several of the most common ENT-related oral infections are further described.

### 3.1. Candidiasis

One of the most common oral infections, especially amongst HIV-positive individuals and the elderly population, is oral candidiasis [5,20,24,31,32,33]. Also known as “thrush” or “candidosis”, this fungal infection is characterized by *Candida* spp. overgrowth and invasion into superficial tissue layers, subsequently damaging the oral mucosal surface [4].

Amongst *Candida* species, *Candida albicans* is considered the primary causative pathogen of oral candidiasis [5,27,34,35]. This is due to its high capability for adherence to oral tissues and denture surfaces, resulting in biofilm formation [35,36]. Nonetheless, this oral infection may also be caused by non-*albicans Candida* species such as *C. glabrata*, *C. guillermondii*, *C. krusei*, *C. parapsilosis*, *C. pseudotropicalis*, *C. stellatoidea*, *C. tropicalis*, *C. keyfr*, and *C. dubliniensis*, which are prevalent and important opportunistic pathogens in immunocompromised patients [5,20,35,37,38,39,40,41].

**Figure 2:**
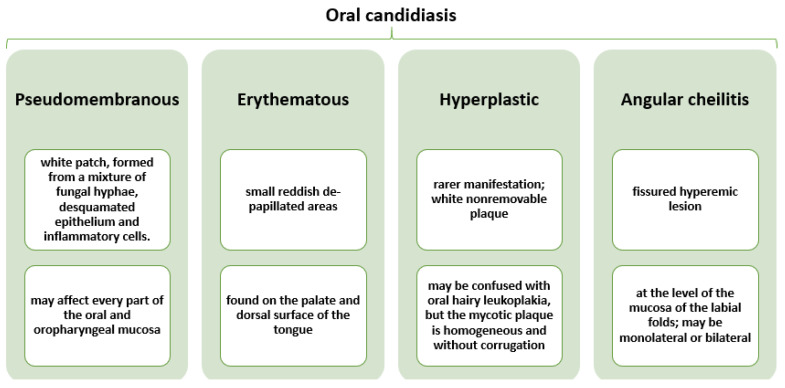
Characterization of different forms of oral candidiasis. Created based on information from reference [32].

Candidiasis can occur at the level of oropharynx, hypopharynx, and larynx, usually producing severe odynophagia and swallowing difficulties [31]. However, 60% of oral candidiasis has been reported in the oral and pharyngeal region in a population of healthy, ambulatory, and immunocompetent individuals [20], while fungal laryngeal infection is rather uncommon, being more frequently observed in immunocompromised patients or individuals with mechanical, chemical, or thermal injuries to the mucosal barrier [25]. Candidiasis may appear in four different forms [32], as described in Figure 2.

The accumulation of pathogens on the host’s mucous membranes, acrylic surfaces of removable orthodontic devices, and denture prostheses leads to the production of proteolytic enzymes that damage mucosal cells [12]. Thus, a dangerous focus of inflammation is created that increases the risk of cerebral strokes, decompensated glycemia, and focal and autoimmune diseases [27].

To prevent the occurrence of systemic *Candida* infections, prophylaxis treatment against this fungus may be provided to patients at risk. Nonetheless, this must proceed with care, as in stem cell transplant recipients and hematological malignancies a microbiota imbalance may be shifted towards the overgrowth of *Aspergillus* and other molds that produce dangerous fungal infections instead [42].

### 3.2. Aspergillosis

Aspergillosis represents the second most common type of opportunistic fungal infection after candidiasis [20,43,44,45]. As the name suggests, this oral mycosis is caused by *Aspergillus* spp., with the most frequently identified species being *Aspergillus fumigatus*, *Aspergillus flavus*, *Aspergillus niger*, and *Aspergillus terreus* [20,45].

The main exposure route to this pathogen is through the inhalation of spores that commonly colonize the upper and lower respiratory tracts. Hence, *Aspergillus* spp. first causes rhinosinusitis and broncho-pulmonary infections that may further spread to the skin, orbits, nose, larynx, and palate [44,45,46]. Tissue invasion is uncommon in immunocompetent individuals, but life-threatening complications can occur in patients with HIV infection, hematological malignancies, diabetes mellitus, or drug-induced immunosuppressive states [20,43,44,46]. This happens because in healthy people acquiring this infection the inhaled fungus is destroyed by macrophages and neutrophils, whereas in immunocompromised patients microorganisms may pass through without being intercepted due to neutropenia or neutrophil dysfunction [44,45].

Invasive aspergillosis remains a highly lethal form of opportunistic mycosis despite available antifungal therapies and improvements in underlying disease management [43]. Therefore, early detection and treatment are crucial in avoiding severe complications [44].

### 3.3. Herpes

The oral herpes viruses HSV-1 and HSV-2 are very common and infectious DNA viruses that persist in the host organism, reactivating periodically due to stress or immunosuppression [24,47,48]. The viral activity is manifested through the appearance of small, painful ulcerations that are most often located in the mouth, hard palate, gums, and on the lips and skin around the mouth, which can coalesce to form giant herpetic lesions [16,31].

Generally, HSV-1 is associated with orofacial infections, swollen lymph nodes, fever, muscle aches, and encephalitis, while HSV-2 is primarily connected to genital infections [16,24,49]. However, HSV-2 infection may spread to the mouth through oral sex, thus producing oral herpes [16].

One of the most frequently reported clinical manifestations of primary HSV infection is primary herpetic gingivostomatitis (PHGS), which occurs in 25–30% of affected children. The pain associated with PHGS disturbs food and water intake, sleep, physical well-being, and the psychological status of both patients and family [50].

HSV can form persistent, long-term, latent infections in sensory neurons and produce lesions at the entry point of the human body [49]. Oral herpes viruses debilitate patients and affect oral health, also having an important psychological impact [47,50]. These viruses have been reported in connection with diseases like diabetes, cancer, myocardial infarction, and Alzheimer’s disease [24,51]. With increasing seroprevalence rates, HSV infection treatment is challenging, irrespective of the various available drugs. Specifically, long-term treatment with antiviral formulations is associated with toxicities and drug resistance. Therefore, new antiviral therapies must be developed [49].

### 3.4. Cytomegalovirus

Cytomegalovirus is a member of the *Herpesviridae* family that can infect many tissues, including salivary glands and deep periodontal pockets [19,52]. Like HPV, cytomegalovirus establishes lifelong latency after the primary infection, manifested through periodical lytic reactivation and viral shedding [53].

This DNA virus can be acquired in two main ways: at mucosal sites through community exposure or by blood-borne transmission [52]. In children, it can cause various conditions such as jaundice, enteritis, central nervous system disturbances, or congenital defects, depending on the age of acquiring the virus. In contrast, adults may present mononucleosis-like symptoms or be completely asymptomatic. Hence, because of the persistent asymptomatic infections it creates, cytomegalovirus can easily be transmitted through the saliva of healthy asymptomatic adult carriers [19].

In immunosuppressed individuals and HIV-positive patients, human cytomegalovirus is a cause of high morbidity and mortality rates. This is due to the small number of available drugs, low potency, poor oral bioavailability, and emergence of drug resistance, which impede the proper treatment of such viral infections [54].

### 3.5. HPV

HPV is a DNA virus belonging to the *Papillomaviridae* family, and infects the skin and mucous membranes [55]. It is one of the most common causes of sexually transmitted infections [56]. Due to the changes in sexual habits in recent decades (e.g., a reduction in the age of onset of sexual activity, an increase in the number of partners, and changes in oro-genital sexual habits), the epidemiological features of HPV infections have also changed, leading to the emergence of an otorhinolaryngological pathology that was rarely seen previously [57,58]. Consequently, many epidemiologists have defined HPV as an endemic infection [58]. Most oral HPV infections are latent or subclinical, spontaneously regressing between 1 and 2 years after the virus is acquired [59].

**Figure 3:**
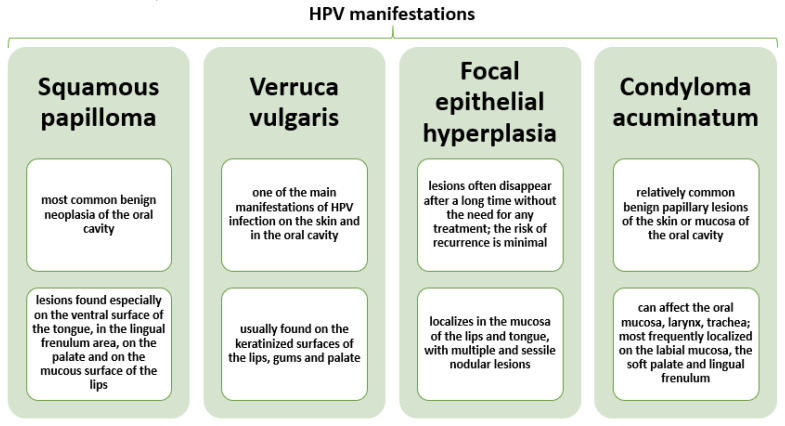
Characterization of most frequent HPV oral clinical manifestations. Created based on information from reference [32].

HPV infections are generally asymptomatic, but they can induce benign tumor formation in some people and cause premalignant lesions that may further develop into cancer [55,60,61]. In the oral mucosa, they have been associated with warts, papilloma, focal epithelial hyperplasia, leucoplakia, oral neoplasia, and condyloma [32,55,58] (Figure 3).

A dramatic increase in the incidence of HPV-induced carcinoma [57,62] has been reported. This virus causes around 70% of all oropharyngeal squamous cell cancer in the United States, with an increased incidence among men which has more than doubled in the past 20 years [63]. Hence there is a growing need to focus on HPV oral diseases and develop efficient prevention and treatment methods [58].

### 3.6. Bacterial Infections

Some of the most common oral pathologies are of bacterial origins, caused by the overgrowth of microorganisms like *Streptococcus mutans*, *Streptococcus salivarius*, *Streptococcus sanguinis*, *Streptococcus aureus*, *Porfiromonas gingivalis*, *Prevotella intermedia*, *Actinobacilus actinomycetemcomitans*, *Enterococcus faecalis*, *Escherichia coli*, *Enterobacter* spp., *Klebsiella* spp., and *Pseudomonas* spp., among others [9,64]. *Streptococcus mitis* is another type of bacteria in the human mouth that is commonly found in the throat and nasopharynx as a colonized organism. It can cause infection in immunocompromised patients with moderate or severe clinical diseases [33]. The risk for oral infections by opportunistic bacteria is also increased in individuals taking chemotherapeutic drugs, as this may alter the receptor interaction between pathogens and epithelial cells and increase bacterial adhesion, while reducing the salivary secretion rate and oral pH. Local factors such as dentures, implants, piercings, wounds, mucositis, and xerostomia have also been reported to contribute to bacterial infection development [64].

Oral mucosal infections can appear either as localized lesions or as generalized stomatitis, with symptoms ranging from almost unnoticeable discomfort to severe pain. In addition, the treatment of bacterial infections in the oral cavity is difficult due to impaired host defense and antibiotic multi-resistance possessed by these pathogens. Hence, prevention is essential and can only be achieved through strict oral hygiene measures [64].

## 4. Synthetic Antimicrobial Drugs for the Treatment of Oral Infections

Depending on the type of oral infection, localization, and aggravating status, several treatment options can be employed.

The most conventional and efficient currently available drugs for treating oral candidiasis are polyenes (e.g., amphotericin B, nystatin), azoles (e.g., miconazole, clotrimazole, fluconazole, itraconazole, voriconazole, posaconazole, ketoconazole), and echinocandins (e.g., anidulafungin, caspofungin, micafungin), which can be administered either locally or systemically. Nonetheless, toxicity, adverse effects, and acquired resistance hinder the use of these antifungals [37,65,66,67].

Similar antifungal agents (i.e., amphotericin B, itraconazole, voriconazole, echinocandins) are also considered for treating aspergillosis in patients with normal immune systems. However, their effect in immunocompromised individuals is not very clear. In addition to antifungal therapy, surgical debridement may be involved [43,46].

Concerning HSV infections, the most accepted therapies imply the use of viral DNA replication inhibitors [68]. The drug of choice for this purpose is acyclovir [16,48,68]. Related nucleoside analogs such as valacyclovir, famciclovir, and ganciclovir may also be involved in the prophylaxis and treatment of HSV infections as they have a similar anti-HSV mechanism [48]. Particularly, valaciclovir has been noted to bring several advantages over acyclovir usage, namely greater oral bioavailability, high plasma levels of the parent compound, greater efficacy, and decreased dosing frequency [16]. Prior to acyclovir’s introduction, the first antiviral for systemic administration was vidarabine. This substance lacks specificity and is more toxic and less metabolically stable than acyclovir, but it is still applied for treating acyclovir-resistant HSV strains [16].

The treatment options are also limited against cytomegalovirus infections, for which the currently used antiviral drugs are ganciclovir, cidofovir, and foscarnet. The best results are obtained when administering the antiviral agents as preemptive treatment (when an asymptomatic cytomegalovirus infection is detected by laboratory analysis) because it helps avoid unnecessary drug toxicity and resistance [54].

Regarding bacterial infections, antibiotics represent the main traditional therapy for microbial control [69,70]. However, the efficiency of antibiotics is hindered by the resistance of slime-like biofilms [69]. Other possibilities for combating bacterial biofilms are bacteriophages and quorum-sensing inhibitors [70].

Table 1 summarizes the most used conventional treatments against oral infections as an overview of the above-presented synthetic antimicrobial drugs.

## 5. Natural Sources of Antimicrobial Compounds

Therapeutic challenges such as adverse effects, low efficiency, and drug resistance developed by numerous pathogens towards conventional treatments have created a need for the development of novel products [71,72,73]. As an alternative to synthetic drugs, antimicrobial compounds from natural sources have gained increasing attention. Whether used to treat the symptoms, subsequential conditions, or the infection itself, natural compounds are of great importance in dealing with oral infections [5,8,10,11,12,54,68,74,75,76].

There are various mechanisms through which bioactive compounds can exert their antimicrobial activity. These include, but are not limited to, destruction of the cell wall or membrane, hindering microbial DNA replication/repair, inhibiting ribosomal protein synthesis, inducing reactive oxygen species production, inhibiting energy synthesis, inhibiting bacterial toxins to the host, inhibiting biofilm formation, reversing antimicrobial resistance, and synergetic effects with antibiotics [77,78].

**Figure 4:**
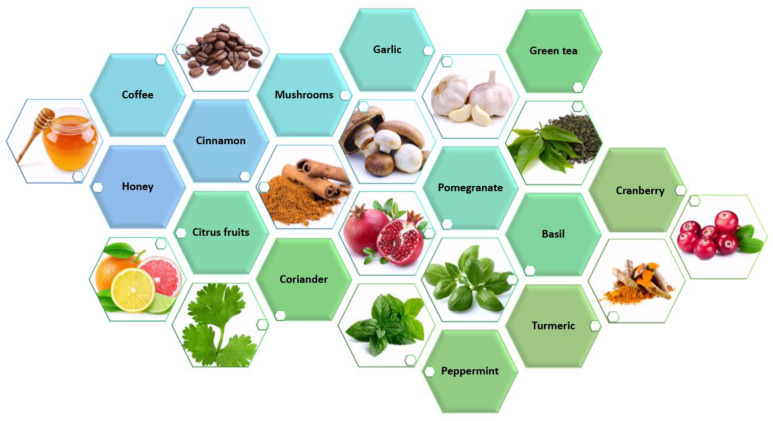
Examples of natural sources of antimicrobial compounds.

In this respect, essential oils, extracts, juices, and pure compounds from various natural sources (Figure 4) have been investigated for their antifungal, antibacterial, antiviral, and antibiofilm activities.

### 5.1. Plant-Derived Natural Compounds

Cinnamon (*Cinnamomum zeylanicum*), a widely used culinary ingredient, has also found applications in medicine, being studied during pregnancy, for diabetes control, and for gynecological problems. Features of interest for oral infections, such as anti-inflammatory, antioxidative, and antimicrobial properties, have also been investigated. It was reported that cinnamon essential oils, extracts, and pure compounds have antibacterial and antifungal properties that can be exploited in the development of mouth rinses, toothpaste, or root canal irrigating solutions. However, as the antifungal activity was observed to be more pronounced than the antibacterial potential, cinnamon could serve as the main or complementary agent in treating candidiasis [9]. Particularly, the effects of mouthwash and spray containing cinnamon essential oil on *Candida* spp have been analyzed. A reduction of 61% and 33% of fungi isolates from oral mucosa and dentures, respectively, was noted, whereas the participants of the study reported a pleasant taste and only a few product-related complaints [79].

Turmeric (*Curcuma longa*) is an evergreen herb endowed with many pharmacological properties of interest for oral infection therapies. Its chloroform extract contains sesquiterpenes, turpentine, and fatty acids that are linked to overall antibiofilm activity. Notably, sesquiterpenes have the ability to destroy bacterial cell membranes due to their lipophilicity, which affects the growth and metabolism of bacteria [70]. Curcumin, the major constituent of turmeric, is rather investigated for its antifungal properties, as it displays potent activity against *C. albicans*, *Aspergillus* spp., *Paracoccidioides brasiliensis*, and *Cryptococcus neoformans*. Nonetheless, curcumin was reported to also be effective against bacteria such as *Streptococcus pyogenes* (at a median MIC of 31.25 µg/mL), methicillin-sensitive *S. aureus* (250 µg/mL), *Acinetobacter lwoffii* (250 µg/mL), and individual strains of *Enterococcus faecalis* (62.5 µg/mL) and *Pseudomonas aeruginosa* (62.5 µg/mL). Furthermore, curcumin can attain antibiofilm activity by inhibiting bacterial quorum-sensing (QS) systems and removing already existing biofilms. The mechanisms of action against microbial strains include induction of the apoptosis pathways and photodynamic action via production of cytotoxic reactive oxygen species against both planktonic and biofilm forms. Hence, these bioactive compounds are promising constituents of new medications with superior performance and fewer adverse effects [80,81].

Green tea (*Camelia sinensis*) is an important natural source of multi-purpose antimicrobial phytochemicals [5]. The aqueous extract of green tea can decrease the number of viable fungal cells in biofilms formed on acrylic resin [82]. Specifically, *C. sinensis* has shown remarkable antifungal activity against *Candida* spp., for example *C. albicans* (at an MIC of 0.125 µg/mL), *C. parapsilosis* (0.125 µg/mL), *C. tropicalis* (0.125–0.250 µg/mL), and *C. glabrata* (0.125–0.250 µg/mL) [83]. Anti-infectious properties have also been demonstrated for tannins isolated from *C. sinensis* extract [84]. In addition, green tea is a valuable source of polyphenols that endow this plant with antioxidant and antiviral properties. Polyphenols can inhibit enzymes that damage cell membranes and prevent the binding and penetration of viruses to cells [85]. Moreover, tea polyphenols have the ability to modify odorant sulfur components, thus abolishing bad breath (halitosis) [5].

Citrus fruits represent a rich source of phytochemicals with many benefits for human health. Possessing numerous therapeutic properties such as anticancer, antiviral, antitumor, antioxidant, and anti-inflammatory activities, citrus fruits have also attracted interest for preventing and treating oral infections [86]. Particularly, limonene, a monocyclic monoterpene found in the rind of citrus fruits, has been shown to have strong anti-biofilm activity against *S. mutans* (~75% biofilm inhibition at a concentration of 400 µg/mL), when used as a coating on oral implants [87]. Additionally, limonene interferes with the growth of yeast cells, being able to inhibit pathogens such as *C. albicans*, *C. krusei*, *C. glabrata*, and *C. parapsilosis* [88].

Peppermint (*Mentha piperita*) is another herbal remedy that finds applications for diverse symptoms and diseases. It has been recognized to have antiseptic, antibacterial, and antifungal properties [89]. Peppermint essential oil was shown to inhibit *C. albicans* and *C. dubliniensis* biofilm formation at a concentration of a maximum of 2 μL/mL in a dose-dependent manner. The antifungal effect is induced by the high concentration of menthol, which can enter the fungal cell membrane and disrupt it. Through this action mechanism, peppermint is also efficient against azole-resistant strains [90].

Castor oil plant (*Ricinus communis*) is an alternative for the creation of antifungal root canal irrigating solutions, mouthwashes, sanitizers, and toothbrushes for complete dentures. Studies compared the effectiveness of castor oil with that of conventional drugs, with the results showing similar potency to miconazole [91]. Another study evaluated and compared the antimicrobial activity of leaf, stem, and root extracts. It was reported that, at a 500 μg/mL concentration, the ethanol extract of the leaves presented antibacterial activity against *P. aeruginosa* and antifungal activity against *C. albicans*. The ethanol extract of the roots was effective against *P. aeruginosa* and *C. glabrata*, while the ethanol extract of the stems only inhibited *P. aeruginosa*. The ethyl acetate extract of the leaves had bacteriostatic activity against *S. aureus*, whereas the hexane extract of the roots exhibited antibacterial effects against *B. subtilis* [92]. Hence, *R. communis* may be used for denture stomatitis treatment, improving the clinical status of elderly patients [91].

Pomegranate (*Punica granatum*) bark extract has an antifungal activity that can also be exploited for treating denture stomatitis. *P. granatum* also presents antiviral, antioxidant, anti-inflammatory, and anti-carcinogenic properties, which are attractive features in creating pharmaceutical formulations against oral infections [91]. Moreover, pomegranate peel is rich in polyphenols responsible for its broad antimicrobial activity against both Gram-positive and Gram-negative bacteria, including methicillin-resistant *S. aureus.* Specifically, for the latter-mentioned pathogen, methanol peel extract was seen effective at a concentration as low as 12 μg/mL, with an inhibition zone of 12.5 mm [93].

Basil (*Ocimum basilicum*) extracts were investigated against *Candida* spp. adhesion on acrylic surfaces of removable orthodontic appliances. It was reported that two extracts (i.e., ethyl acetate and *n*-hexane fraction) were able to inhibit the growth, adherence, and formation of *C. albicans* and *C. dubliniensis* biofilms in a proportion of 73% and 78%, respectively, in the vicinity of ethyl acetate fractions, and 65% and 78%, respectively, in the vicinity of the *n*-hexane fraction. Therefore, they can be included in antifungal solutions or mouthwashes that can prevent and treat oral *Candida* infections [12].

Another natural anti-*Candida* treatment may be based on coriander (*Coriandrum sativum*) essential oil. This essential oil showed similar inhibitory activity to nystatin against *Candida* spp. planktonic cells and *C. albicans* biofilm (0.125 mg/mL for *C. albicans* CBS 562 and 1 mg/mL for *C. albicans* clinical isolate 13A5). Hence, this plant has a promising potential for oral candidiasis prevention and treatment [90,94].

Horsetail (*Equisetum giganteum*) also exhibits antifungal properties. When added to denture fixative powder, this plant’s hydroethanolic extracts influenced *C. albicans* biofilm formation on acrylic surfaces, minimizing its colonization and reducing its metabolism [91]. At a concentration of 16 mg/mL, an up to 79% reduction in biofilm cell viability was reported 24 h after treatment [95]. Moreover, horsetail antimicrobial effects were also reported against other pathogens such *as Streptococcus pyogenes*, *Bacillus cereus*, *Bacillus subtilis*, *Enterococcus faecalis*, *Staphylococcus aureus*, and *Staphylococcus epidermidis* [91].

Cranberry (*Vaccinium macrocarpon*) represents another natural source of interest against oral bacterial species. Cranberry juice can inhibit acid production, attachment, and biofilm formation, and even reverse microbial co-aggregation in the form of high molecular weight non-dialysable material (NDM) [96]. Thus, it is considered a promising preventive measure to include an NDM fraction in toothpastes and mouthwashes to better control oral diseases [97,98]. The most abundant flavonoids extracted from these fruits, proanthocyanidins (PACs), have been shown particular antimicrobial, antiadhesion, anti-inflammatory, and antioxidative properties [99]. A recent study specifically investigated PAC activity against *P. aeruginosa*. It was reported that PACs extracted from cranberry inhibit biofilm formation by 40.9% and 55.7%, at concentrations of 1 μg/mL and 10 μg/mL, respectively. These flavonoids were also shown to reduce preformed biofilm by 54.1% (*p* < 0.05) at 10 μg/mL concentration, and by 39.6% at (*p* <  0.01) at a concentration of 100 μg/mL [100].

Resveratrol is another important compound from cranberry. This polyphenolic antioxidant can be also found in peanuts (*Arachis hypogea*), blueberries (*Vaccinium* spp.), Japanese knotweed (*Polygonum cuspidatum*), and grapevines (*Vitis vinifera*). Resveratrol demonstrated antimicrobial activity against bacteria and fungi such as *B. cereus* (at an MIC of 50 μg/mL), *S. aureus* (100–512 μg/mL), *E. faecalis* (100–342 μg/mL), *M. smegmatis* (64 μg/mL), *S. pneumoniae* (100 μg/mL), *S. pyogenes* (>200 μg/mL), *E. coli* (250—512 μg/mL), *K. pneumoniae* (250–512 μg/mL), *P. aeruginosa* (200–512 μg/mL), and *C. albicans* (20–300 μg/mL). Its mechanisms of action against microbial strains include inhibition of ATP hydrolysis and synthesis, DNA fragmentation, and membrane damage due to increased potassium leakage and increased propidium iodide uptake [101].

Antibacterial activity was reported for garlic (*Allium sativum*) extract as well. Specifically, the active component allicin has been shown to permeate the bacterial membrane, destroy the cell structure, change the gene expression of microorganisms, and react with thiol enzymes to induce oxidative stress [70,102]. As allicin is a volatile compound, researchers had the idea to test its efficiency in the gas phase. It was reported that most of *Pseudomonas*, *Streptococcus*, and *Staphylococcus* isolates were completely inhibited by allicin at a 64 μg/mL concentration [103]. Moreover, antifungal properties were also noted, as a crude extract of 49 μg/mL concentration inhibited the growth of *C. albicans* [77].

Summer savory (*Satureja hortensis*) is also of interest for developing pharmaceuticals and natural therapies for infectious diseases. Its essential oil has been tested against 23 bacteria and 15 fungi species, showing great antimicrobial potential. Contrastingly, methanol extracts were not as efficient; only the nonpolar subfraction was reported to have antibacterial activity against five bacterial species, namely *Bacillus subtilis* (250 μg/mL), *Enterococcus faecalis* (500 μg/mL), *Pseudomonas aeruginosa* (250 μg/mL), *Salmonella enteritidis* (500 μg/mL), and *Streptococcus pyogenes* (500 μg/mL) [104].

The lavender tree (*Heteropyxis natalensis*) is traditionally used for oral care. The ethanolic extract of its leaves and twigs was investigated for antimicrobial activity against several oral microorganisms, of which *Actinomyces israelii* was found to be the most sensitive (at an MIC of 0.88 mg/mL). *H. natalensis* can also reduce the acid produced by *S. mutans* and *L. paracasei*, diminishing the metabolic effects of cavity-causing bacteria while only moderately influencing commensal microorganisms. Hence, this plant’s extract may be used for preventing excessive tissue damage in oral diseases by reducing pro-inflammation [15].

Tasmanian blue gum (*Eucalyptus globulus*) leaves were also reported to have antibacterial activity against oral bacteria (e.g., *P. gingivalis*, *S. mutans*). Including 0.4–0.6% eucalyptus extract in chewing gum significantly contributed to the inhibition of plaque formation, inflammation, and gingiva bleeding [105,106].

Gum Arabic tree (*Acacia nilotica*) has been used in ancient medicine for treating a broad range of diseases (e.g., abdominal aches, sore throat, dysentery, asthma, diabetes, hypertension). The plant’s twig has also gained attention for dental care due to its phytochemical content; fractions of *A. nilotica* twig methanol extract presented inhibitory properties against selected oral pathogens (zones of inhibition in the range 14–40 mm). The most potent effect was obtained for *E. faecalis*, with an MIC of 80 μg/mL. In addition, the identified bioactive compounds (e.g., catechins, catechol, gallic acid, sitosterol, kaempferol, etc.) can be included in herbal toothpaste, endodontic irrigating solutions, mouth fresheners, mouthwashes, and dental gels for maintaining healthy oral microflora [30].

Baikal skullcap (*Scutellaria baicalensis*) has attracted interest for various therapeutic purposes. Baicalein, the most important compound from this plant’s root extract, has antimicrobial, antioxidant, anticancer, and anti-inflammatory activities, which can be applied for treating several diseases. In addition, these naturally occurring anti-biofilm compounds are considered promising for novel strategies in combating pathogenic bacteria and treating biofilm-associated infections [11]. For instance, over 70% inhibition of *C. albicans* biofilms was registered for concentrations between 4 and 32 μg/mL [107].

Another natural source of antimicrobial, antiviral, and anti-inflammatory compounds is almond (*Prunus dulcis*) skin. Researchers have created a mix of polyphenols present in natural almond skin which displays anti-herpetic pharmacological properties that can be exploited for designing topical formulations. Nonetheless, further studies must be performed in order to establish possible synergistic effects with currently approved antibiotics and antivirals [68].

A similar anti-herpetic action was indicated for polyphenols extracted from natural shelled pistachios (*Pistacia vera*) kernels. Thus, pistachio extracts could serve as a novel treatment for HSV-1 infections, either alone or combined with standard antiviral therapies. Moreover, the antiviral and anti-inflammatory properties suggest possible further interest in using pistachio product waste as a source of bioactive compounds for pharmaceutical formulations [48].

One polyphenol of particular importance is tannic acid. Its unique antiviral and antibacterial properties have attracted interest in developing new strategies for preventing and treating oral infections. Tannic acid presents significant antimicrobial activity against influenza A virus, papillomaviruses, noroviruses, HSV-1, HSV-2, and HIV, as well as activity against both Gram-positive and Gram-negative bacteria without the toxicity associated with classic drugs [84].

The collateral effects of oral infections can also be diminished using plant-derived natural compounds. For instance, *Echinacea purpurea* extract has been investigated for sore throat therapy [105]. It was noticed that the effects on the sore throat of a sage/echinacea spray were comparable with those of chlorhexidine/lidocaine, with 60% of the patients in each group becoming symptom-free after 3 days [108].

Coconut oil is an alternative therapeutic option, especially in irradiated head and neck cancer patients. Coconut oil can “coat” the mouth and form a barrier that maintains the moisture of mucosal surfaces. Thus, it represents a feasible, low-cost, and safe strategy for managing xerostomia, which is a common complication of many diseases and represents a burden on patients’ quality of life [28].

### 5.2. Honey and Beehive Products

The medicinal properties of honey and beehive products have attracted interest for use in otorhinolaryngology. In addition, the immunomodulatory and antimicrobial properties of honey, propolis, royal jelly, and bee pollen are useful for diverse applications [109].

Honey has antibacterial, antiviral, and anti-inflammatory impacts, a low toxicity profile, and wound healing-enhancing properties [50,110]. Its mechanisms of action include hyperosmolarity, low pH, production of hydrogen peroxide, and a unique composition containing antioxidant compounds [110]. Particularly, in patients who underwent radio- and chemotherapy of the oropharyngeal region, honey was shown to reduce the intensity of oral mucositis, *Candida* infection, and pathogenic bacteria, while allowing faster healing [50,109]. Furthermore, the antiviral activity of honey was studied as an alternative to synthetic drugs for treating herpes lesions. A study compared honey versus acyclovir topical application. By analyzing factors such as healing time, pain relief, resolution of local signs, and duration of acute attacks, it was concluded that honey is superior to synthetic products by 43%, 39%, 28%, and 35%, respectively. These findings were attributed to the copper, ascorbic acid, and hydrogen peroxide contents of honey, which can inactivate HSV [111,112]. A particularly appealing type of honey for antimicrobial applications is Manuka honey, which has been shown effective in preventing biofilm growth and reducing acid production. Its antimicrobial potency is related to the Unique Manuka Factor (UMF) rating, which depends on the methylglycoxal and total phenol content [113,114,115]. The effects of several types of honey are compared in Table 2.

Propolis is a non-toxic, antimicrobial, anticancer, antibiotic, antifungal, antiviral, and anti-inflammatory natural product [5,116]. These biological and curative properties drew attention for inhibiting biofilm formation and treatment of denture stomatitis [91]. In particular, propolis extracts can be included in medicinal products, mouthwashes, toothpaste, and dental varnishes to control the growth of *Candida* spp. [8]. Research has shown that red propolis alcoholic extract exerts fungistatic and fungicidal activity on *C. albicans* (at 32–64 μg/mL and 64–512 μg/mL, respectively), *C. tropicalis* (32–64 μg/mL and 64 μg/mL), and *C. glabrata* (64 μg/mL and 64–256 μg/mL) strains isolated from chronic periodontitis cases [117]. An in vivo study on patients with full dentures demonstrated the anti-*Candida* activity of a mouthrinse based on a hydroalcoholic extract of propolis. The yeast strains showed antifungal activity in the following order of decreasing sensitivity: *C. albicans*, *C. tropicalis*, *C. krusei*, and *C. guilliermondii* [118].

Royal jelly, the yellow-white creamy and acidic secretion produced by worker honeybees to feed the queen honeybee, has attracted interest due to its composition rich in minerals, vitamins, fatty acids, sugars, proteins, and free amino acids. It has been observed to have immunomodulatory, wound-healing, bacteriostatic, antioxidant, and antimicrobial properties against yeasts and Gram-negative and Gram-positive bacteria [119,120,121]. Royal jelly administration showed promising results in patients undergoing radio- and chemotherapy, improving the signs and symptoms of oral mucositis and shortening the healing time [122]. Royal jelly has also been tested for the treatment of herpetic lesions as a natural alternative to acyclovir, showing inhibitory effects on HSV-1 at 250 μg/mL concentration [123].

Bee pollen is another bee product presenting useful pharmacological properties such as antifungal, antimicrobial, antiviral, anti-inflammatory, and immunostimulating activity. Its ethanol extract has been shown to be effective against *S. aureus*, *E. coli*, *K. pneumoniae*, *P. aeruginosa*, and *C. albicans*, becoming an interesting alternative for preventing and managing oral infections [124].

### 5.3. Other Natural Sources and Compounds

Mushrooms do not fall into any of the above-presented categories, but their health benefits are important to be mentioned in the context of alternative antimicrobial therapies. Mushrooms have bioactive compounds that have been shown to present antiviral properties. They contain polysaccharides, carbohydrate-binding proteins (i.e., polysaccharopeptide, peptidomannan), proteins (i.e., ubiquitin-like protein, nebrodeolysin, lectin, lentin), peptides, enzymes (i.e., laccase, tyrosinase), polyphenols, triterpenes, triterpenoids, and several other compounds that can inhibit viral entry, replication, viral enzymes, and the expression of viral proteins and cellular proteins. Antiviral compounds from mushrooms can enhance the immune system, helping the organism fight against HSV-1, HSV-2, HIV, and the influenza A virus, among others. Hence, mushroom-derived bioactive metabolites could serve as antiviral candidates against DNA and RNA viruses [125].

Various compounds possessing important antibacterial and antibiofilm activities have also been obtained from microalgae. Ethanolic extracts of *Chorella vulgaris* and *Dunaliella salina* are promising for inhibiting bacterial biofilm formation. Specifically, the compounds responsible for the antimicrobial properties may be flavonoids, tannins, and terpenoids from *C. vulgaris* extract and 3,3,5-trimethylheptane, *n*-hexadecane, polyunsaturated fatty acids, β-ionone, and neophytadiene from *D. salina* extract [126].

Natural polysaccharides extracted from cyanobacteria and macroalgae have been established as potent antiviral compounds that can disturb virus–cell interactions and inhibit virus adsorption or penetration into the host cells [49,54]. From *Nostoc flagelliform* nostoflan can be isolated, which is an antiviral polysaccharide that can act against cytomegalovirus infections. With an even higher potency against human cytomegalovirus than nostoflan, calcium spirulan can be used in alternative natural therapies. This sulfated polysaccharide isolated from *Spirulina platensis* owes its antiviral properties to the presence of sulfated groups, but further investigations are needed to clarify its structural formula [54]. Algal polysaccharides also have promising activity against influenza B virus and mumps, as is the case of compounds obtained from *Gelidium cartilagineum* (Linnaeus) Gaillon. Sulfated polysaccharides present in algae can also be employed in the development of novel HSV infection therapies; however, so far, only a small number of species have been investigated for anti-HSV properties [49]. The rich content in bioactive compounds of seaweeds has attracted interest in their antimicrobial potential as well. Hence, algae can be used for developing cost-effective therapies with only minor toxicity and fewer secondary effects than synthetic antibiotics [10].

A group of natural compounds that have attracted research interest towards infection treatment is represented by antimicrobial peptides (AMPs) [127]. Particularly, AMPs have been investigated for the control of bacterial biofilms [8]. AMPs are part of the line of defense against pathogens in higher organisms, but in microorganisms they compete for nutrients. Thus, natural AMPs are relatively safe and well-tolerated by humans, also being highly effective. However, in antifungal therapy only a few peptides were employed. Their use is hindered by hemolytic activity, low bioavailability, a poor ability to cross physiological barriers, and loss of activity in high salt concentrations [128].

To determine a clear correlation between the natural sources, therapeutic properties, inhibited pathogens, responsible bioactive compounds, and potential applications in treating oral diseases, Table 3 was created.

## 6. Hybrid Treatment Options

Some oral pathogens have also developed resistance to single-plant extracts [5], thus creating the need for the development of hybrid treatments.

One option is to create novel formulations that combine several plants [5]. An example of such mixture is made from *Azadirachta indica*, *Mangifera indica* L., *Hemidesmus indicus* (L.) R.Br., *Caryophyllus aromaticus* L., *Cinnamomum zeylanicum* Blume, *Quercus infectoria* Oliv., *Emblica officinalis* Gaertn., *Terminalia belerica* Roxb., and *Terminalia chebula* Retz. The chewable poly-herbal tablet was shown to inhibit the growth of dental bacteria, demonstrating good antimicrobial activity [143]. Another medicinal plant mixture is Sho-Saiko-to. Each of its seven herbal components (i.e., *Bupleureum falcatum*, *Glycyrrhiza uralensis*, *Panax ginseng*, *Pinelliae ternatae*, *Scutellaria baicalens*, *Zingiber officinale*, and *Ziziphus jujube*) contain several active biochemical constituents which work in synergy to treat oral infections [5,144].

Another possibility is the design of antimicrobial drugs by combining the benefits of natural compounds with the advantages of nanotechnology [145]. One successful association is the nanostructure made of polylactic acid-based composite films embedded with magnetite nanoparticles conjugated in situ with *Eucalyptus* essential oil. The inorganic material has a role in stabilizing and potentiating the essential oil, while the polymer modulates the biocompatibility and stability of magnetite. Such coatings do not affect the viability of eukaryote cells, but they significantly interfere with the formation and maturation of bacterial biofilms. Hence, effective anti-infective therapeutic nanosystems are obtained that can offer targeted and controlled treatment [146].

Studies combining silver nanoparticles with algae extracts have also shown promising results [126]. For instance, silver nanoparticles containing *Oscillatoria* spp. green algae methanol extract exhibit strong antibiofilm and antibacterial activity against pathogens such as *S. aureus*, *E. coli*, *P. aeruginosa*, and *B. cereus*. Their enhanced performance, coupled with their low cytotoxicity, are important criteria for their potential use in pharmacological applications [147].

Hydrogels embedded with tannic acid-modified silver nanoparticles were tested against herpes virus infection and were shown to be effective. These nanostructures affect viral attachment, impede penetration, and reduce post-infection spread [84,148].

The synergic action of natural compounds and conventional drugs represents another hybrid treatment option. It was reported that the combination between curcumin and antibiotics could restore the sensitivity of bacteria to synthetic drugs. Hence, it diminishes bacterial toxicity while promoting the influx of antibiotics [70]. Regarding viral infections, the combined use of acyclovir and honey resulted in better outcomes than the antifungal drug used alone in treating PHGS; specifically, children presented a significant earlier disappearance of oral lesions (3 days vs. 6 days), drooling (2 days vs. 4 days), and eating difficulty (3 days vs. 8 days). Moreover, honey improved inflammation and decreased the associated pain, allowing the patients to maintain a normal diet and recover from the infection [50].

To provide an overview of the status of hybrid treatment options, Table 4 summarizes several examples of patents in the field of oral care products.

## 7. The Role of Diet and Nutrition in Preventing Oral Infections

Like any form of life, microorganisms need nutrients to live and grow. The host’s diet can also be their source of nourishment, influencing the number and types of microbes in the oral cavity [19]. Therefore, by controlling the diet, some oral infections can be prevented.

A deficiency in micronutrients (e.g., B vitamins) is associated with oral manifestations like glossitis, cheilitis, and angular stomatitis. It has also been noticed that undernutrition aggravates oral infections, contributing to life-threatening conditions such as noma, a dehumanizing oro-facial type of gangrene [160].

On the other hand, the ingestion of high levels of carbohydrates is not beneficial either, as microorganisms use them as their primary energy source. Hence, a large preponderance of microbes is seen in individuals consuming large amounts of refined sugar and those drinking beer [5,19].

A healthy diet should be rich in fruits, vegetables, and wholegrain starchy foods, while the intake levels of free sugars and fat should be as low as possible [160]. The ingestion of bioactive natural compounds promotes oral health through their negative immunoregulatory and anti-inflammatory activities [161]. Everyday widely available products like edible mushrooms, honey, green tea, cranberries, grapes, milk, coffee, and alcohol-free red wine are natural foods and beverages proven to inhibit bacterial adhesion in the oral cavity [5].

Therefore, maintaining a balanced diet is an important factor in preventing oral diseases and infection invasion.

## 8. Conclusions and Future Perspectives

To conclude, oral infections are a hot topic of research, especially due to the emerging drug resistance to conventional treatments, contamination and invasion potential, and psychological impact. The toxicity, adverse effects, and low efficacy of existing synthetic drugs have driven the exploration of natural alternatives. Many compounds and products derived from plants, algae, fungi, and other natural sources have been investigated for their antimicrobial properties, leading to promising results against oral-related pathogens. Hence, it can be expected that novel natural pharmaceutical formulations and oral hygiene products will soon emerge on the market.

However, the tested species represent only a small part of the thousands of sources available in nature. Hence, research efforts should be directed towards the other useful plants that remained unexplored. Moreover, the mechanisms of action of some of the discussed natural antimicrobial compounds have not yet been fully elucidated, requiring additional investigations. Particularly, the selective targeting ability towards pathogens instead of probiotics should be evaluated for the discussed natural-based treatment alternatives. Another problem to be solved in the near future is assessment of the toxicological safety of the extracts and pure compounds that have been only tested in vitro so far. Ultimately, close attention should be given to match the treatment with the causative pathogen and the customized needs of each individual for the development of personalized anti-infective therapies.

## Figures and Tables

**Table 1:** The most commonly used synthetic antimicrobial drugs for treating oral infections.

Oral Infection	Conventional Treatment	Main Limitations	Refs.
Candidiasis	Amphotericin B, clotrimazole, miconazole, nystatin, itraconazole, ketoconazole, fluconazole	Toxicity, adverse effects, drug resistance	[37,65,66,67]
Aspergillosis	Amphotericin B, itraconazole, voriconazole, echinocandins	Toxicity, adverse effects, interaction with other drugs	[43,46]
HSV	Acyclovir, valacyclovir, famciclovir, ganciclovir, vidarabine	Toxicity, drug resistance	[16,31,48]
Cytomegalovirus	Ganciclovir, cidofovir, foscarnet	Toxicity, drug resistance	[54]
Bacterial infections	Antibiotics	Drug resistance	[69,70]

**Table 2:** Comparison of the effects of different types and concentrations of honey on *S. aureus*. Reprinted with permission from ref. [115]. Copyright 2017 Elsevier B.V.

Concentration	Sample	Broth Dilution Method		Agar Dilution Method	
		Mean Methicillin-Sensitive *S. aureus* (CFU/mL)	Mean Methicillin-Resistant *S. aureus* (CFU/mL)	Mean Methicillin-Sensitive *S. aureus* (CFU/mL)	Mean Methicillin-Resistant *S. aureus* (CFU/mL)
-	Control	3.40 × 10^7^	5.50 × 10^6^	2.03 × 10^8^	3.90 × 10^8^
10% (*v*/*v*)	Manuka + 10	3.70 × 10^3^ *	5.50 × 10^3^ *	3.75 × 10^4^ *	4.55 × 10^5^ *
	Manuka + 16	4.00 × 10^1^ *	5.05 × 10^2^ *	4.19 × 10^4^ *	5.15 × 10^4^ *
	Manuka + 20	0.33 × 10^1^ *	0.50 × 10^1^ *	2.10 × 10^2^ *	1.19 × 10^3^ *
	*Nigella sativa*	3.70 × 10^6^	5.50 × 10 ^5^	1.55 × 10^8^	3.90 × 10^8^
	Sidr	3.67 × 10^5^	1.00 × 10^5^	1.55 × 10^8^	3.90 × 10^8^
20% (*v*/*v*)	Manuka + 10	4.00 × 10^1^ *	5.00 × 10^2^	1.02 × 10^4^ *	1.00 × 10^2^ *
	Manuka + 16	0.33 × 10^1^ *	5.00 × 10^2^	2.50 × 10^2^ *	0.00 *
	Manuka + 20	0.00 *	0.00 *	1.00 × 10^1^ *	0.00 *
	*Nigella sativa*	7.00 × 10^4^ *	5.50 × 10^4^	7.27 × 10^4^ *	8.60 × 10^7^
	Sidr	3.67 × 10^4^ *	1.00 × 10^5^	9.97 × 10^7^	1.50 × 10^8^
50% (*v*/*v*)	Manuka + 10	0.00 *	0.00 *	0.00 *	0.00 *
	Manuka + 16	0.00 *	0.00 *	0.00 *	0.00 *
	Manuka + 20	0.00 *	0.00 *	0.00 *	0.00 *
	*Nigella sativa*	0.00 *	0.00 *	0.00 *	0.00 *
	Sidr	0.00 *	0.00 *	0.00 *	0.00 *

* The differences between control and tested honey were significant at the 0.05 level.

**Table 3:** Correlation between natural antimicrobial compounds, microorganisms, oral diseases, and potential applications.

Natural Source	Form	Bioactive Compounds	Therapeutic Properties and Effects	Pathogens against Which Activity Was Reported	Oral Disease	Potential Applications	Refs.
Cinnamon	Essential oil, extracts, pure compounds	Trans-cinnamaldehyde, cinnamate, cinnamic acid	Anti-inflammatory, cardioprotective, antioxidant, antimicrobial, antibacterial, antifungal	*Candida* spp., *E. coli*, *P. gingivalis*, *B. cereus*, *S. aureus*, *S. epidermidis*, *S. pyogenes*, *Pseudomonas* spp., *Salmonella* sp.	Oral candidiasis, bacterial infections	Mouth rinses, mouthwash, spray, toothpaste, root canal irrigating solution	[9,79,129]
Turmeric	Chloroform extract	Curcumin, sesquiterpenes, turpentine, fatty acids	Antifungal, antioxidant, antibacterial, antibiofilm	*C. albicans*, *Aspergillus* spp., *Paracoccidioides brasiliensis*, *Cryptococcus neoformans*	Bacterial infections and biofilms, oral candidiasis, aspergillosis	New medications with fewer side-effects	[70,80]
Green tea	Aqueous extract, powder, semi-fermented, non-fermented	Polyphenols, tannins	Antimicrobial, antifungal, antiviral, anti-infectious, antioxidant, inhibits growth, adherence, and formation of bacterial biofilm	*C. albicans*, *C. parapsilosis*, *C. Tropicalis*, *C. Glabrata*, *S. mutans*, *P. gingivalis*, *Actinobacillus actinomycetemcomitans*, *Prevotella intermedia*, *S. mitis*, *S. sanguis*	Oral candidiasis, halitosis, bacterial biofilms	Tea, mouthwash, chewing gum, mouth spray	[5,69,82,83,84,85]
Citrus fruits	Essential oil, extracts, juice	Limonene, alkaloids, flavonoids	Anticancer, antiviral, antitumor, antioxidant, anti-inflammatory, antibiofilm, antibacterial, antifungal	*S. mutans*, *C. albicans*, *C. krusei*, *C. glabrata*, *C. parapsilosis*	Oral candidiasis, bacterial biofilms	Coating oral implants, inclusion in daily diet	[86,87,88,130]
Peppermint	Essential oil	Menthol	Antimicrobial, antifungal, antibacterial, antiseptic, antispasmodic	*C. albicans*, *C. dubliniensis*, *S. aureus*	Oral candidiasis, bacterial infections	Potentiator for existing antibiotics	[89,90]
Castor oil plant	Oil	Ricinoleic acid	Antifungal, analgesic, anti-inflammatory, antimicrobial	*C. albicans*, *E. faecalis*	Denture stomatitis	Root canal irrigating solution, toothbrush for complete dentures, mouthwash, sanitizer	[91,131,132]
Pomegranate	Bark extract, peel extracts	Polyphenols, tannins, flavonoids	Antimicrobial, antifungal, antiviral, antioxidant, anti-inflammatory, anticancer	*Aspergillus* spp., *Candida* spp., *Salmonella* spp, *E. coli*, *E. faecalis*, *S. aureus*, *S. mutans*, *B. subtilis*	Denture stomatitis, aspergillosis, bacterial infections	Pharmaceutical formulations	[91,93]
Basil	Extracts, essential oil	Linalool	Antifungal, antimicrobial, antioxidant, inhibits growth, adherence ad formation of biofilm	*C. albicans*, *C. duliniensis*, *S. aureus*, *S. saprophyticus*, *E. coli*	Fungal and bacterial biofilms	Antifungal solution, mouthwash, potentiator for existing antibiotics	[12,89,133]
Coriander	Essential oil, extracts	2-hexen-1-ol, 3-hexen-1-ol, cyclodecane	Inhibitory activity	*Candida* spp.	Oral candidiasis, fungal biofilms	Natural antifungal formulations,	[90,94]
Horsetail	Hydroethanolic extracts	Phenolic compounds, flavonoid heterosides	Antimicrobial, antifungal, antibiofilm, anti-inflammatory	*S. pyogenes*, *B. cereus*, *B. subtilis*, *E. faecalis*, *S. aureus*, *S. epidermidis*, *C. albicans*	Oral candidiasis, denture stomatitis, fungal and bacterial biofilms	Additive for denture fixative powder, topical formulations	[91,134]
Cranberry	Juice, pure compounds	Proanthocyanidins (PACs), resveratrol	Antimicrobial, antibacterial, antibiofilm, antiadhesion, anti-inflammatory, antioxidative	*S. mutans*, *E. coli*, *P. aeruginosa*, influenza virus	Bacterial infections, bacterial biofilm	Toothpaste, mouthwashes	[96,97,98,99,100]
Garlic	Extracts, oil	Allicin, ajoene, diallyl trisulfide, allyl alcohol, diallyl disulfide	Antibacterial, antimicrobial, antiviral, antifungal, antiprotozoal	*Candida* spp., *Aspergillus* spp., *Cryptococcus* spp., *Pseudomonas* spp., *Proteus* spp., *S. aureus*, *E. coli*, *B. subtilis*, *Salmonella* spp., *Klebsiella* spp., cytomegalovirus, HSV, HIV	Bacterial infections, HSV infections, fungal biofilms	Pharmaceutical formulations (alone or in combinations with conventional antibiotics)	[70,102]
Summer savory	Essential oil, nonpolar subfraction of the methanol extract	Carvacrol, thymol, γ-terpinene, p-cymene	Antimicrobial, antispasmodic, antioxidant, sedative	*B. subtilis*, *E. faecalis*, *P. aeruginosa*, *S. enteritidis*, *S. pyogenes*	Bacterial infections	Pharmaceutical formulations, natural therapies, tea, additives in food	[104]
Lavender tree	Ethanolic extract, essential oil	Cardamomin, aurentiacin A, quercetin, 3,5,7,-trihydroxyflavan, 5-hydroxy-7-methoxyflavanone	Antimicrobial, antibacterial, antifungal	*A. israelii*, *S. mutans*, *L. paracasei*, *S. aureus*, *P. aeruginosa*, *Aspergillus* spp.	Bacterial infections	Tea, pharmaceutical formulations	[15,97]
Tasmanian blue gum	Extract, essential oil	1,8-cineole, linalool, pinocarveol	Antioxidant, antibacterial, inhibit plaque formation, inflammation, and bleeding of gingiva	*S. mutans*, *F. nucleatum*, *P. gingivalis*, *S. aureus*, *E. coli*, *K. pneumoniae*	Bacterial infections	Pharmaceutical formulations, toothpaste, mouthwash, additive for chewing gum	[105,135,136]
Gum Arabic tree	Extracts	Catechins, catechol, gallic acid, sitosterol, kaempferol, niloticane, D-pinitol, linoleic acid	Antimicrobial, antibacterial, antifungal, antioxidant, anticancer	*E. faecalis*, *S. aureus*, *S. mutans*, *C. albicans*, *K. pneumoniae*, *B. subtilis*, *B. cereus*	Bacterial infections, fungal biofilms	Herbal toothpaste, endodontic irrigating solutions, mouth fresheners, mouthwashes, dental gels	[30,137]
Baikal skullcap	Root extracts	Baicalein	Antimicrobial, antioxidant, anticancer, anti-inflammatory, antibiofilm, antiviral	*P. aeruginosa*, *S. saprophyticus*	Bacterial infections, bacterial biofilms	Pharmaceutical formulations	[11,138,139]
Almond	Skin extract	Polyphenols	Anti-inflammatory, anti-herpetic, antimicrobial	HSV-1, *S. aureus*	HSV-1 infections	Topical formulations	[68]
Pistachio	Extracts (water, chloroform, ethanol)	Polyphenols	Anti-inflammatory, antiviral, inhibits growth, adhesion, biofilm formation and acid-producing ability of bacteria	HSV-1, *S. mutans*, *S. salivarious*, *S. sobrinus*, *S sanguis*	HSV-1 infections, bacterial biofilms	Topical or oral formulations (alone or in combination with standard antiviral therapies)	[48,69]
Echinacea	Extracts	Cichoric acid, caffeic acid, alkamides, polysaccharides	Antiviral, antibacterial, anti-inflammatory, immune-modulatory, antioxidant	*C. albicans*, *S. pyogenes*, *H. influenzae*, *L. pneumophila*, HSV-1, HSV-2, HIV	Sore throat, tonsilitis, bacterial infections, herpes	Spray, pharmaceutical formulations	[105,108,140]
Coconut	Virgin oil	Medium chain fatty acids	Antimicrobial, antibacterial, antifungal, antiviral, antibiofilm	*S. aureus*, *E. coli*, *S. enteritidis*, *B. cereus*, *P aeruginosa*, *S. mutans*	Xerostomia, bacterial biofilms	Treatment strategy for irradiated head and neck cancer patients, dietary supplement	[28,141]
Honey	As such	Phenolic compounds, amino acids, enzymes, Maillard reaction products	Antimicrobial, antifungal, antibacterial, antiviral, anti-inflammatory, antioxidant, immune-modulatory, wound healing	*Candida* spp., HSV	Oral mucositis, oral candidiasis, herpes	Topical application, dietary supplement	[109,110,111]
Propolis	Hydroalcoholic extract	Flavonoids	Antimicrobial, anticancer, antifungal, antiviral, anti-inflammatory, antibiotic, immune-modulatory, inhibits biofilm formation	*C. albicans*, *C. tropicalis*, *C. krusei*, *C. guilliermondii*, *C. glabrata*	Oral candidiasis, denture stomatitis, fungal biofilms	Mouthwash, mouthrinse, toothpaste, dental varnishes	[5,8,91,116,117,118]
Royal jelly	Raw or purified product	Royalisisn, trans-10-hydroxy-2-decenoic acid, jelleines, apalbumins, apolipophorin-III-like protein, glucose oxidase	Antimicrobial, antioxidant, antibacterial, immune-modulatory, wound healing	*S. aureus*, *S. epidermidis*, *E. faecalis*, *P aeruginosa*	Oral mucositis, herpes, bacterial infections	Pharmaceutical formulations, dietary supplement	[119,120,121]
Bee pollen	Ethanol extract	Flavonoids, phenolic acids, fatty acids	Antifungal, antimicrobial, antiviral, anti-inflammatory, immune-modulatory, anticancer, local analgesic, wound healing	*S. aureus*, *E. coli*, *K. pneumoniae*, *P. aeruginosa*, *C. albicans*	Bacterial infections	Topical application, dietary supplement	[124,142]
Mushrooms	Extracts	Polysaccharides, carbohydrate-binding proteins, proteins, peptides, enzymes polyphenols, triterpenes, triterpenoids	Antimicrobial, antiviral	HSV-1, HSV-2, influenza A virus, HIV	HSV infections	Pharmaceutical formulations	[125]
Algae	Extracts, pure compounds	Sulfated polysaccharides	Antimicrobial, antibacterial, antibiofilm, antiviral, antitumor, anticoagulant	Influenza B virus, mumps, HSV	HSV infections, bacterial biofilms	Antimicrobial therapies with less secondary effects	[10,49,126]

**Table 4:** Examples of patented applications of oral care products based on natural compounds.

Oral Care Product	Natural Sources and Forms	Bioactive Compounds	Other Active Compounds in the Product	Claimed Therapeutic Properties and Effects	Patent/Patent Application Number	Refs.
Oral care composition for topical application	Cranberry extract	Pronathocyanidins	Sodium cocoyl glutamate Vegetable glycerin Sodium monofluorophosphate Bioactive glass (calcium sodium phophosilicate) Soy lecithin Carrageenan and xanthan gum	Reduction of plaque build-up on teeth Inhibition of bacteria to the gums	US8715625 B1	[149]
Oral care composition in the form of a gel	Magnolia extract Hops extract	Honokiol Magnolol Hexahydrogenated beta acids	Sodium saccharin Sodium fluoride Tetrasodium pyrophosphate Sodium tripolyphosphate Glycerin Sorbitol	Antibacterial Anti-inflammatory Antiplaque Antigingivitis	US 8900644 B2	[150]
Toothpaste oral rinse	Seed or pulp extract of *Citrus* and *Vitis* plant families	Polyphenols	Potassium nitrate Metal cations salts Polyphosphates Pyrophosphates Phosphonates Fluoride ion source Xylitol	Prevention or treatment of halitosis Antimicrobial effect	US 6,706,256 B2	[151,152]
Oral hygiene composition	Grape seed aqueous extract	Polyphenols, mainly oligo-proanthocyanin	Inorganic fluorine salts	Anti-biofilm effect Reduced microbial colonization	US 2010/0129297 A1	[151,153]
Oral rinse and mouthwash	Essential oils (e.g., eucalyptol, menthol, methyl salicylate, thymol, tea tree oil, peppermint, spearmint, clove) Grape seed extract *Citrus* seed extract Immunostimulant selected from *Echinacea*, goldenseal, hawthorne berry, myrrh, rosehips, *Lomatium dissectum*, *Astragalus* root, licorice root	Polyphenols	Hydrogen peroxide Alcohol	Antimicrobial Anti-inflammatory Soothing effect	US 8,273,385 B1	[151,154]
Oral hygiene tablets and capsules	Bioflavonoids from citrus fruits Skin extract of red grapes Turmeric rhizome *Boswellia serrata* Fennel seed	Flavonoids Anthocyanins	Glycerin Sodium bicarbonate Hydrogen peroxide Fluoride	Anti-inflammatory Soothing effect Protective effect on gums and mouth tissue	US 8,728,446 B2	[151,155]
Gargle tablet	*Citrus* extract	Citric acid	Chlorhexidine acetate Sodium carbonate Sodium bicarbonate Sorbitol	Antibacterial Anti-inflammatory Prevention or treatment of halitosis	CN 1306814 A	[156]
Medicine in the form of oral tablet	Crude gallnut extract	Gallotannin	Polyvinylpyrrolidone Polyethylene glycol	Antibacterial Accelerated healing of oral ulcers Prevention or treatment of halitosis	CN 102228479 B	[157,158]
Toothpaste, alcohol-free mouthwash, and whitening wand	Essential oils (e.g., lemon oil, lime oil, sweet orange oil, ginger oil, tea tree oil, wintergreen oil, spearmint oil, peppermint oil, ylang ylang oil, vanilla oil, cinnamon oil, clove oil, grapefruit oil, eucalyptus oil, myrrh oil) Coconut oil	Phenols Tannins	Xylitol Calcium citrate Diatomaceous Earth Malic acid Xanthan gum Potassium sorbate Vitamins Minerals	Antimicrobial Soothing effect Maintenance of a balanced pH and neutralization of acids produced by bacteria Removal of plaque	US 20190175956 A1	[159]

## Data Availability

Not applicable.
